# Impact of hypogonadism on bone mineral density and vertebral fractures in HIV-infected men

**DOI:** 10.1007/s40618-021-01665-7

**Published:** 2021-08-30

**Authors:** L. C. Pezzaioli, T. Porcelli, A. Delbarba, F. Maffezzoni, E. Focà, F. Castelli, C. Cappelli, A. Ferlin, M. E. Quiros-Roldan

**Affiliations:** 1 Department of Clinical and Experimental Sciences, Unit of Endocrinology and Metabolism, University of Brescia and ASST Spedali Civili Brescia, Viale Europa 11, 25123 Brescia, Italy; 2 Endocrinology, Montichiari Hospital, ASST Spedali Civili Brescia, Montichiari (Brescia), Italy; 3 Department of Infectious and Tropical Diseases, University of Brescia and ASST Spedali Civili Hospital, Brescia, Italy

**Keywords:** HIV, Vertebral fractures, Osteoporosis, Hypogonadism, SHBG, FSH

## Abstract

**Purpose:**

Hypogonadism and osteoporosis are frequently reported in HIV-infected men and, besides multifactorial pathogenesis, they might be directly linked because of testicular involvement in bone health. We evaluated the prevalence of osteoporosis and vertebral fractures (VFs) in HIV-infected men, and assessed their relationship with gonadal function.

**Methods:**

We enrolled 168 HIV-infected men (median age 53). Osteoporosis and osteopenia were defined with T-score ≤  – 2.5SD and T-score between  – 1 and  – 2.5SD, respectively. VFs were assessed by quantitative morphometric analysis. Total testosterone (TT), calculated free testosterone (cFT), Sex Hormone Binding Globulin (SHBG), Luteinizing Hormone (LH) and Follicle Stimulating Hormone (FSH) were obtained; overt hypogonadism was defined on symptoms and low TT or cFT, and classified into primary and secondary according to gonadotropins; compensated hypogonadism was defined as normal TT and cFT with high LH levels.

**Results:**

Overall, osteoporosis and osteopenia were found in 87.5% of patients, and VFs were detected in 25% of them; hypogonadism was identified in 26.2% of cases. Osteoporotic patients had higher SHBG *vs* those with normal bone mineral density (BMD). Fractured patients were more frequently hypogonadal and with higher SHBG. SHBG showed negative correlation with both spine and femoral BMD, and positive correlation with VFs. In multivariate models, FSH showed negative impact only on femoral BMD, whereas older age and higher SHBG predicted VFs.

**Conclusion:**

We found a high burden of bone disease and hypogonadism in HIV-infected men, and we showed that the impact of gonadal function on bone health is more evident on VFs than on BMD.

## Introduction

The introduction of combined antiretroviral therapy (cART) has radically changed the course of HIV infection with an increase in life expectancy, and has therefore, led to an increased prevalence of aging-related diseases, such as hypogonadism and osteoporosis [1]. In addition, HIV infection itself is associated with chronic inflammation and premature aging [2], and HIV + patients experience more aging-associated comorbidities and at an earlier age compared to non-infected counterparts [3].

Hypogonadism results from failure of the testis to produce physiological amounts of testosterone and/or spermatozoa and might depend on alteration at one or more levels of the hypothalamic-pituitary–testicular (HPT) axis [4]. The diagnosis of hypogonadism relies on the combination of low morning testosterone levels and clinical manifestations of androgen deficiency [4, 5], including sexual symptoms (reduced libido, erectile dysfunction, decreased spontaneous erections) and less specific signs and symptoms (loss of body/facial hair, decreased testicular volume, increased body fat/reduced muscle mass, central obesity, osteoporosis, asthenia, decreased concentration, etc.) [4]. However, the diagnosis of hypogonadism in men with HIV can be challenging, as they often present with non-specific symptoms or with signs/symptoms overlapping with other comorbidities (e.g., obesity, HCV, diabetes) [1]. Furthermore, sexual symptoms in men with HIV might have multiple causes other than hypogonadism [1].

Osteoporosis is also frequent in HIV-infected subjects [1], who have lower spine and hip bone mineral density (BMD), with a three-fold risk of osteoporosis *vs* controls [6]. Although data on vertebral fractures (VFs) are less conclusive, previous metanalyses showed a two-folds increased risk of VFs compared to non-infected patients [7, 8]. Importantly, whether low BMD leads to more fractures in HIV-infected patients is still debated [7]. Both hypogonadism and osteoporosis in HIV-infected men have a multifactorial pathogenesis [1, 9], including smoking, alcohol use, physical inactivity, cART effect and chronic immune activation by HIV infection itself [10]. Furthermore, hypogonadism and osteoporosis might be directly associated [1], since testicular function is involved in bone health [11]. In fact, Leydig cells play a role in bone health by producing not only testosterone, but also insulin-like 3 peptide (INSL3), and by contributing to the 25-hydroxylation of vitamin D [12], which may be all involved in BMD maintenance [13]. Reduced testicular function is a known cause of male osteoporosis at all ages also in general population [14]. However, data on the association between testosterone and BMD in HIV-infected males are not conclusive, and data on hypogonadism and VFs in these patients are very scarce. Furthermore, methodologic discrepancies in the assessment of hypogonadism exist among previous studies, and no study with fractures as primary endpoint has been performed [1].

We, therefore, aimed to investigate the prevalence of osteoporosis and VFs in a cohort of HIV-infected men under cART, and to assess their relationship with properly investigated gonadal function.

## Methods

### Patients and methods

A cross-sectional retrospective study on 168 HIV-infected males was performed. Inclusion criteria were: age > 18 years, serologically documented HIV infection in stable condition under cART, no personal history of malabsorption or drugs with potential detrimental effect on bone, blood samples carried out at central hospital laboratory, DXA scan and quantitative morphometric assay performed with the same densitometer (Explorer Hologic Inc., QDR-4500 W Waltham, MA) according to normative Italian data [15].

DXA results were expressed as BMD (g/cm^2^) and were collected for both spine, total hip and femoral neck. For patients older than 50 years, T score (standard deviations -SD- above or below sex and ethnicity matched population at peak of bone mass) was calculated. According to guidelines, for patients younger than 50 years, both T and Z scores were collected, being Z score SD above or below sex and ethnicity matched population of the same age. However, taking into account the very small number of patients younger than 50 years, and to allow comparison between densitometric parameters, we included only T score for statistical analysis. A T score less than or equal to  – 2.5 SD in at least one site (spine or hip) was defined as osteoporosis, whereas T score values between  – 1 and  – 2.5 SD at both spine and hip sites were diagnostic for osteopenia [16].

VFs were assessed through a quantitative morphometric assay performed on images obtained from DXA by the same operator and by measuring anterior, middle and posterior vertebral heights and height ratios for each vertebra from T5 to L4 [17].

Hypogonadism was defined based on the concurrent presence of suggestive signs/symptoms (reduced libido, morning erections, erectile function) and biochemical findings of low total testosterone (TT) and/or calculated free testosterone (cFT) levels, and subsequently classified according to luteinizing hormone (LH) levels into primary (high LH) and secondary (low/normal LH). cFT was calculated with Vermeulen equation (http://www.issam.ch/freetesto.htm), that combine TT, sex hormone-binding globulin (SHBG) and albumin. Cut-off value for lower limit of TT and cFT were 3.46 ng/ml and ≤ 65 pg/ml, respectively [4, 5]. Normal range for LH was established between 1.5 and 9.4 mUI/ml [4, 5]. Elevated LH with normal TT and cFT levels identified compensated hypogonadism. More details on hypogonadism assessment in this population are shown elsewhere [18].

All blood samples were obtained between 8.00 and 10.00 a.m., after a 12-h overnight fast. Blood and urinary samples were collected for the following biochemical assays: HBV (chemiluminescence microparticle immunoassay—CMIA) and HCV (chemiluminescence immunoassay—CLIA) serostatus, CD4 count (flow cytometry), TT (CMIA), SHBG (CLIA), follicle stimulating hormone (FSH) (CMIA), LH (CMIA), serum and urinary calcium and phosphate, parathyroid hormone (PTH) (CLIA), 25-OH-vitamin D (High-Pressure Liquid Chromatography with UV Detector – HPLC–UV), bone-alkaline phosphatase (affinity electrophoresis), C-terminal telopeptide (Enzyme-linked immunosorbent assay—ELISA), osteocalcin (CLIA). Intra- and inter-assay coefficient of variance for TT, LH and SHBG was < 5%.

Information on smoking and drinking habits were collected. Patients were defined as current smokers if actively smoking at least 1 cigarette/day, and past smokers if they had quit smoking for at least one year. We considered patients as usual drinkers if they drink alcohol at least once a day, and occasional drinkers if they drink once a week or less.

Ethical approval for this study was obtained from Local Ethical Committee (Comitato Etico di Brescia, NP 3898) and informed consent was obtained from all participants.

### Statistical analysis

Statistical Package for the Social Sciences software IBM SPSS Statistics, Version 25.0, Armonk, (NY) was used for statistical analysis. Since the variables were not normally distributed (Kolmogorov–Smirnov test was used), comparison between medians of the quantitative variables was performed with non-parametric Kruskal–Wallis H test (followed by post hoc Bonferroni test when a significant difference was found) or Mann–Whitney U test, as appropriate. Comparison between categorial variables was performed with Pearson’s Chi Square. Correlation between bone parameters and clinical and biochemical data was performed with Spearman or point-biserial correlation, as appropriate. To investigate factors associated with BMD, multivariate models were designed, using hierarchical multiple regression analysis. To assess predictive factors for VFs, a backward stepwise logistic regression analysis was performed. All multiple regression analyses were preceded by univariate analyses to identify candidate predictive variables. Receiver operating characteristic (ROC) curves were used to study sensitivity and specificity of FSH, testosterone and SHBG in identifying patients with normal BMD from those with osteopenia/osteoporosis and with and without VFs. Area under the curve (AUC) < 0.5 would not discriminate affected and non-affected patients. *P* values < 0.05 were considered statistically significant.

## Results

Characteristics of the study participants, according to bone mineral status and to VFs, are shown in Table 1. Median age of the whole population was 53 years (IQR 49–57). Only 21/168 patients (12.5%) had normal BMD, whereas 89/168 (53.0%) had osteopenia, and 58/168 (34.5%) had osteoporosis. In 42/168 (25.0%) of the patients, at least one VF was detected. Of them, 22/42 (52.4%) were osteoporotic, 19/42 (45.2%) had osteopenia, and one of them (2.4%) had normal BMD. Regarding gonadal function, 44/168 (26.2%) patients were diagnosed with hypogonadism, including overt (low TT or cFT) and compensated forms (normal testosterone with high LH). Of them, 21/44 (47.8%) had overt hypogonadism: 5/21 (23.8%) had primary and 16/21 (76.2%) had secondary/normogonadotropic hypogonadism. Besides antiretroviral therapy, 9/168 patients (5.4%) were taking calcium supplementation, and 91/168 (54.2%) were already taking adequate vitamin D supplementation.

**Table 1:** Comparison between patients with normal BMD, osteopenia and osteoporosis, and between patients with vertebral fractures vs patients without vertebral fractures

Total (*n*. 168)	Normal values	Normal BMD (*n*. 21)	Osteopenia (*n*. 89)	Osteoporosis (*n*. 58)	*P* value	No vertebral fractures (*n*. 126)	Vertebral fractures (*n*. 42)	*P* value
Age (years)		53 (46–60)	54 (49–57)	52 (49–57)	0.840	52 (48–57)	55 (51–60)	0.048
BMI (kg/m^2^)		25.2 (22.7–28.7)*	25.7 (23–27.9)*	22.7 (20.8–25.9)**	0.001	25.0 (22.3–27.2)	24.6 (21.1–27.1)	0.477
*Smoke*								
Current smoker, # (%)		6/18 (33.3)	29/72 (40.3)	30/44 (68.2)	0.027	45/100 (45)	20/34 (58.8)	0.271
Past smoker, # (%)		3/18 (16.7)	11/72 (15.3)	2/44 (4.5)		14/100 (14)	2/34 (5.9)	
*Alcohol*								
Usual drinker, # (%)		3/18 (16.7)	21/68 (30.9)	12/44 (27.3)	0.715	25/95 (26.3)	11/35 (31.4)	0.785
Occasional drinker, # (%)		10/18 (55.6)	30/68 (44.1)	18/44 (40.9)		44/95 (46.3)	14/35 (40)	
Drug user, # (%)		5/20 (25)	20/77 (26)	27/51 (52.9)	0.004	34/111 (30.6)	18/37 (48.6)	0.047
HIV infection duration (years)		11 (6–20.5)*	11.5 (6–20)*	17 (10.5–24.5)**	0.035	11 (8–20)	18 (9–25)	0.015
cART duration (years)		11 (5–16.5)	8.5 (5–16.8)	13 (8.8–18)	0.087	10 (7–16)	14 (7.5–18)	0.002
CD4 + nadir (cell/mm^3^)		190 (62–325.5)	173 (55.5–287.5)	120 (52–248)	0.523	150 (59–292)	127.5 (48.5–223)	0.300
CD4 + nadir (%)		18.9 (11.6–22.9)	14.9 (8.7–22.1)	14 (7.7–22.6)	0.533	14.9 (8.3–22.2)	15.2 (9.7–22.1)	0.987
CD4 + at inclusion (cell/mm^3^)	470–1240	731 (534–960)	675 (457–799)	560 (412–755.5)	0.160	643 (442–808)	596 (381.3–886.5)	0.697
CD4 + at inclusion (%)	31.0–58.0	32.8 (24.9–38)	31.8 (25.5–37.9)	29.6 (21.7–34.9)	0.326	31.5 (23.9–37.8)	32.1 (25.2–35.7)	0.799
AIDS, # (%)		5/21 (23.8)	24/81 (29.6)	16/52 (30.8)	0.834	31/116 (26.7)	14/38 (36.8)	0.234
HBsAg + , # (%)		1/21 (4.8)	5/79 (6.3)	5/54 (9.3)	0.732	8/116 (6.9)	3/38 (7.9)	0.836
HCV Ab + , # (%)		7/21 (33.3)	27/81 (33.3)	30/52 (57.7)	0.015	44/116 (37.9)	20/38 (52.6)	0.111
Diabetic, # (%)		3/21 (14.3)	12/89 (13.5)	8/58 (13.8)	0.995	16/126 (12.7)	7/42 (16.7)	0.515
Chronic kidney disease, # (%)		1/21 (4.8)	6/89 (6.7)	4/58 (6.9)	0.939	7/126 (5.6)	4/42 (9.5)	0.368
Hypogonadism, # (%)		4/19 (21.1)	22/83 (26.5)	18/53 (34)	0.482	29/119 (24.4)	15/36 (41.2)	0.044
*Type of hypogonadism*								
Primary, # (%)		1/4 (25)	2/21 (9.5)	2/16 (12.5)	0.764	4/28 (14.3)	1/13 (7.7)	0.526
Secondary, # (%)		1/4 (25)	10/21 (47.6)	5/16 (31.3)		12/28 (42.9)	4/13 (30.8)	
Compensated, # (%)		2/4 (50)	9/21 (42.8)	9/16 (56.3)		12/28 (42.9)	8/13 (61.5)	
TT (ng/ml)	3–9	6.2 (4.8–7.5)	6.6 (5.2–8.5)	7 (5.3–9.3)	0.241	6.6 (5.2–8.4)	6.9 (5.1–9.1)	0.438
SHBG (nmol/l)	10–70	49 (41.2–60.5)*	64 (43.5–92)	69.5 (51.8–94.5)*	0.034	59.0 (42.7–73.0)	84.5 (56.8–113.8)	0.010
cFT (pg/ml)	65–260	98.9 (80.6–118)	93.6 (73.6–119.3)	94.3 (71.9–117.8)	0.859	94.9 (74.1–115.3)	88.6 (72.0–119.0)	0.825
LH (IU/l)	1.5–9.0	3.6 (2.9–7.9)	6.3 (3.6–8.2)	6 (4–13.5)	0.079	5.2 (3.3–8.2)	7.5 (4.1–10.7)	0.085
FSH (IU/l)	1.5–8	5.2 (4.1–6.2)	6.9 (3.8–10.3)	6.8 (4.9–11.3)	0.220	5.9 (4.1–9.5)	6.8 (4.7–12.7)	0.322
Calcium, serum (mg/dl)	8.6–10.2	9.3 (8.8–9.7)	9.2 (9–9.6)	9.4 (9.2–9.6)	0.204	9.3 (9.0–9.7)	9.3 (9.1–9.5)	0.994
Calcium, 24 h urine (mg/24 h)	100–300	226.6 (163.5–314)	221 (156–327)	212.5 (122.6–297.8)	0.570	223.3 (158.0–315.0)	201.5 (141.5–300.5)	0.592
Phosphate, serum (mg/dl)	2.5–4.3	2.4 (2.1–2.7)	2.6 (2.2–3)	2.5 (2.2–3.2)	0.283	2.5 (2.2–2.9)	2.8 (2.5–3.3)	0.003
Phosphate, 24 h urine (mg/24 h)	400–1300	816.5 (732.8–1059.5)	910.8 (715.7–1138.5)	810 (578–1066)	0.363	912.5 (730.1–1116.3)	738.0 (552.5–918.0)	0.010
PTH (pg/ml)	11–67	41.8 (32.8–76)	48 (32–67)	51 (39–63)	0.932	45.0 (34.0–70.0)	50.0 (40.8–60.3)	0.774
25-OH-vitamin D (ng/ml)	> 20	32 (26.2–39.5)	28.5 (18.9–38.5)	33 (19.3–42)	0.401	29.5 (19.2–36)	34.5 (23.2–48.7)	0.068
Bone alkaline phosphatase (IU/l)	10–50	32 (22.2–51.8)	34 (27–50.5)	39 (31–60)	0.357	38.0 (29.0–52.0)	32.0 (20.0–58.0)	0.163
C-terminal telopeptide (ng/ml)	0.1–0.7	0.4 (0.2–0.5)	0.5 (0.3–0.6)	0.4 (0.2–0.7)	0.802	0.5 (0.3–0.6)	0.3 (0.2–0.5)	0.266
Osteocalcin (ng/ml)	< 20	9 (5.9–17)	9 (5.3–16.3)	11.4 (7–19.7)	0.380	10.0 (7.0–16.5)	9.0 (5.0–17.8)	0.655
Spine BMD (g/cm^2^)		1.2 (1.1–1.2)**	1 (0.9–1.1)**	0.8 (0.7–0.9)**	< 0.001	1 (0.9–1.1)	0.9 (0.8–1)	0.021
Spine T-score (SD)		0.2 ( – 0.6; 0.5)**	– 1.4 ( – 1.8; – 1)**	– 2.9 ( – 3.5; – 2.7)**	< 0.001	– 1.5 ( – 2.5; – 0.8)	– 2.3 ( – 3; – 1.3)	0.017
Total hip BMD (g/cm^2^)		1.1 (1–1.1)**	0.9 (0.8–1)**	0.8 (0.7–0.9)**	< 0.001	0.9 (0.8–1.1)	0.8 (0.7–0.9)	0.021
Total hip T-score (SD)		0 ( – 0.5; 0.4)**	– 1 ( – 1.5; – 0.3)**	– 1.8 ( – 2.4; – 1.2)**	< 0.001	– 0.9 ( – 1.5; – 0.1)	– 1.5 ( – 2.4; – 1.1)	0.001
Femoral neck BMD (g/cm^2^)		1 (0.9–1) **	0.8 (0.7–0.9)**	0.7 (0.6–0.8)**	< 0.001	0.8 (0.7–0.9)	0.8 (0.7–0.8)	0.091
Femoral neck T-score (SD)		– 0.3 ( – 0.8; 0)**	– 1.3 ( – 1.8; – 1)**	– 2.1 ( – 2.7; – 1.6)**	< 0.001	– 1.2 ( – 1.9; – 0.7)	– 1.8 ( – 2.2; – 1.3)	< 0.001
Vertebral fractures, # (%)		1/21 (4.8)**	19/89 (21.3)**	22/58 (37.9)**	0.006	/	/	/

Variables expressed as median (IQR) or absolute number (%) as appropriate

Comparisons for continuous variables are performed with Kruskal–Wallis, for BMD comparison, and with Mann–Whitney *U* test, for vertebral fractures comparison (post hoc: Bonferroni, identified in table through*). Comparison for categorial variables are performed with Pearson’s Chi Square

A *p*-value ≤ 0.05 is considered significant

Abbreviations: *BMI* body mass index, *cART* combined antiretroviral therapy, *TT* total testosterone, *SHBG* sex hormone-binding protein, *cFT* calculated free testosterone, *LH* luteinizing hormone, *FSH* follicle stimulating hormone, *PTH* parathyroid hormone, *BMD* bone mineral density

Osteoporotic patients had BMI values significantly lower than patients with osteopenia or normal BMD (*p* = 0.001). Furthermore, osteoporotic patients, compared to those with normal BMD or osteopenia, were more frequently active smokers (*p* = 0.027) and drug users (*p* = 0.004), had longer HIV infection duration (*p* = 0.035), were more frequently HCV coinfected (*p* = 0.015), and showed a higher burden of VFs (*p* = 0.006). Finally, osteoporotic patients had higher SHBG values compared to those with normal BMD (*p* = 0.034).

The comparison of patients with and without VFs showed that fractured patients were older (median age 55 vs 52 years, *p* = 0.048), more frequently drug users (*p* = 0.047), had longer HIV infection duration and cART exposure (*p* = 0.015 and *p* = 0.002, respectively), and were more frequently hypogonadal (*p* = 0.044) and with higher SHBG values (*p* = 0.010). BMD data were worse in men with VFs with respect to men without VFs.

**Fig. 1:**
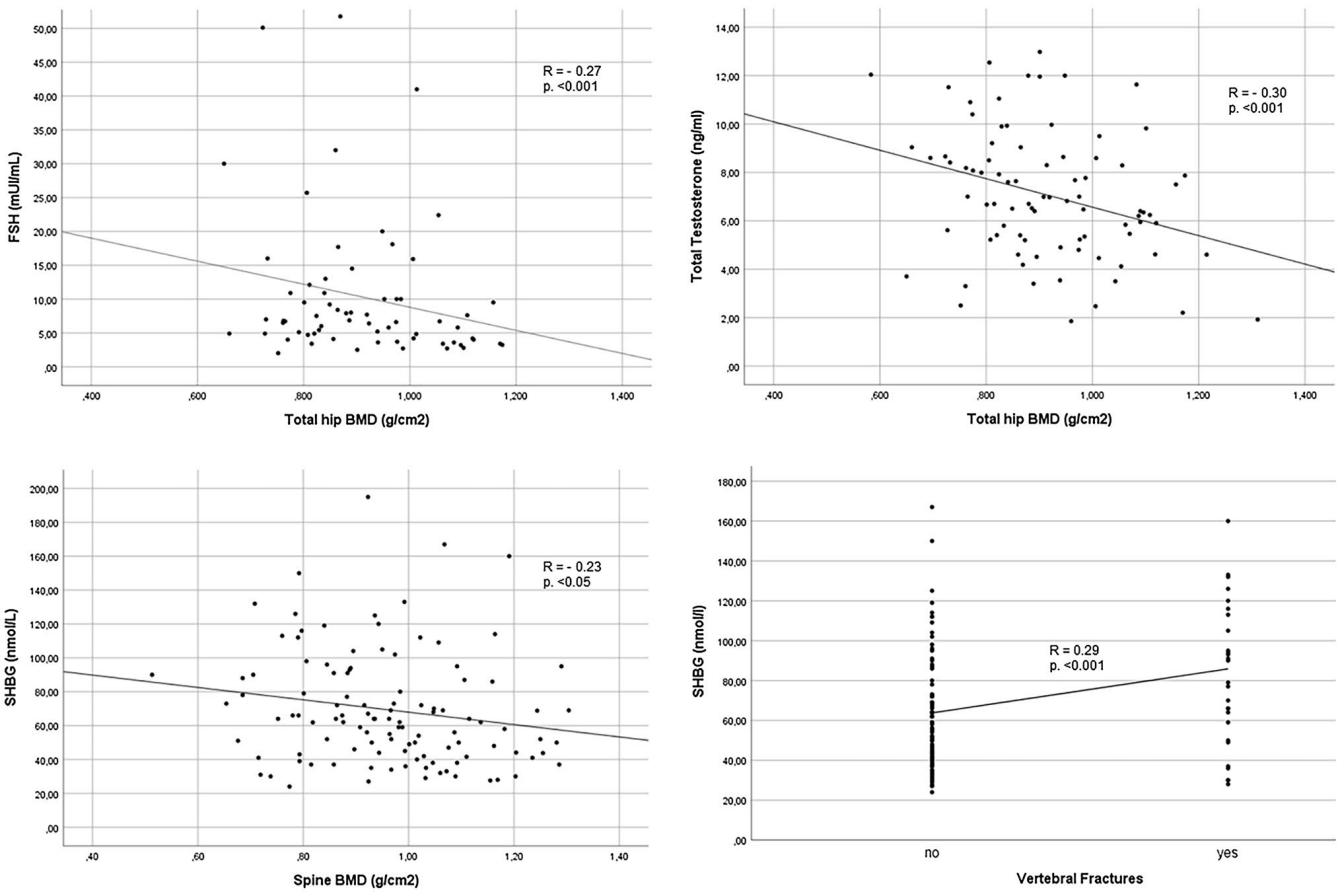
Correlations between gonadal function and BMD/VFs

The relationship between bone mineral status (including BMD, T-score and VFs) and main clinical, virological and biochemical data is shown in Table 2 (and Fig. 1 for gonadal function insight). With respect to BMD data, the strongest positive correlations were between BMI and both spine and femoral BMD and T scores. HIV infection duration was negatively associated with femoral BMD and T scores and with spine T score, and a significant positive correlation was found between total hip BMD and CD4 + count. Focusing on gonadal function, TT levels had moderate-to-strong negative correlation with total hip BMD and with total hip and femoral neck T scores. FSH and LH values showed strong negative correlation with total hip T score, and FSH showed further negative correlation with total hip BMD and femoral neck T score. SHBG levels showed negative correlation with spine BMD, and spine, total hip and femoral neck T scores. With respect to VFs, age, HIV infection and cART duration were positively associated with their presence. Among hormonal data, only SHBG was positively associated with VFs.

**Table 2:** Correlation between bone mineral status/vertebral fractures and main clinical, virological and biochemical data

	Spine BMD	Spine T-score	Total hip BMD	Total hip T-score	Femoral neck BMD	Femoral neck T-score	Vertebral fractures
Age (years)	0.08	0.09	0.04	0.04	0.05	0.05	0.15*
BMI (kg/m2)	0.23**	0.24**	0.37**	0.35**	0.23*	0.32**	– 0.06
HIV infection duration (years)	– 0.12	– 0.16*	– 0.30**	– 0.29**	– 0.08	– 0.15	0.19*
cART duration (years)	- 0.10	– 0.11	– 0.20	– 0.22*	0.01	– 0.10	0.25**
CD4 + (cell/mm^3^) nadir	0.04	0.07	0.22*	0.14	0.10	0.08	– 0.08
CD4 + (cell/mm^3^) at inclusion	– 0.01	0.08	0.22*	0.17	0.17	0.13	0.01
TT (ng/ml)	– 0.11	– 0.11	– 0.30**	– 0.27**	– 0.18	– 0.17*	0.08
SHBG (nmol/l)	– 0.23*	– 0.25**	– 0.22	– 0.29*	– 0.08	– 0.19*	0.29**
cFT (pg/ml)	0.09	0.06	– 0.08	– 0.06	0.05	0.02	– 0.06
LH (IU/l)	– 0.08	– 0.11	– 0.22	– 0.25*	– 0.02	– 0.17	0.10
FSH (IU/l)	– 0.02	– 0.03	– 0.27**	– 0.29**	– 0.14	– 0.21*	0.08
Calcium (mg/dl)	– 0.10	– 0.13	– 0.07	– 0.08	0.06	– 0.10	0.01
25-OH-vitamin D (ng/ml)	0.05	– 0.02	0.02	0.04	0.03	– 0.01	0.15
PTH (pg/ml)	0.01	0.02	0.07	– 0.04	– 0.11	– 0.10	0.03

Spearman correlation was used for continuous-continuous variables correlation; point-biserial correlation was used for continuous-categorical variables correlation

^*^Correlation is significant at *p* = 0.05 (two-tail)

^**^Correlation is significant at *p* = 0.01 (two-tail)

Abbreviations: *BMI* body mass index, *cART* combined antiretroviral therapy, *TT* total testosterone, *SHBG* sex hormone-binding protein, *cFT* calculated free testosterone, *LH* luteinizing hormone, *FSH* follicle stimulating hormone, *PTH* parathyroid hormone, *BMD* bone mineral density

Then, the association between BMD and clinical, virological and biochemical parameters was investigated through univariate and multivariate models. For spine BMD model, no correlation was found at multivariate analysis. In Table 3, the results of hierarchical multiple regression analysis for total hip BMD are shown. We entered variables in four different steps, with each independent variable being assessed for what it might add to the prediction of total hip BMD variance, after the previous variables were controlled for. Once all variables were entered, the overall model was assessed in terms of its ability to predict total hip BMD, and the relative contribution of each set of variables was also reported. In Step 1, we forced age and BMI into the analysis, to controlling for these variables. In this step, age and BMI explained 20.3% of variance associated with hip BMD. In Step 2, we entered data on HIV infection into the model as a block, with the effect of age and BMI removed, to assess whether these variables were still able to predict our dependent variable. We found that CD4 nadir count, HIV and cART duration explained an additional 10.8% of the variance. Following this procedure, Step 3 showed that TT, SHBG, cFT LH and FSH explained a further 10% of variance, controlling for previously entered variables. Finally, in Step 4, VFs alone added another 4.2% of explained variance, controlling for all other factors. The final model (significant at *p* = 0.008) explained 45.3% of the variance associated with total hip BMD. Independent variables with the highest beta’s still significant in the final model for total hip BMD were BMI (β 0.37, *p* = 0.004), TT (β  – 0.38, *p* = 0.032) and FSH (β  – 0.34, *p* = 0.026). With respect to femoral neck BMD, despite the final model being not significant, and therefore, not reported in Table 3, some independent variables showed significant univariate correlation (cART exposure β 0.46, *p* = 0.049; TT β  – 0.49, *p* = 0.014; SHBG β 0.42, *p* = 0.029; cFT β  – 0.30, *p* = 0.038; FSH β  – 0.41, *p* = 0.016; VFs β  – 0.26, *p* = 0.048).

**Table 3:** Hierarchical multiple regression analysis on factors affecting total hip BMD

	Model 1		Model 2		Model 3		Model 4	
	β (st)	Sig	B (st)	Sig	B (st)	Sig	B (st)	Sig
Age (years)	– 0.09	0.453	– 0.11	0.329	– 0.08	0.508	– 0.04	0.721
BMI (kg/m^2^)	0.46	< 0.001	0.45	< 0.001	0.38	0.003	0.37	0.004
HIV infection duration (years)			– 0.46	0.032	– 0.37	0.085	– 0.40	0.059
cART duration (years)			0.35	0.109	0.31	0.137	0.38	0.070
CD4 + (cell/mm^3^) nadir			0.23	0.054	0.20	0.075	0.20	0.078
TT (ng/ml)					– 0.34	0.061	– 0.38	0.032
SHBG (nmol/l)					0.13	0.439	0.23	0.186
cFT (pg/ml)					0.23	0.089	0.22	0.089
FSH (mUI/ml)					– 0.32	0.039	– 0.34	0.026
LH (mUI/ml)					0.05	0.765	0.03	0.831
VFs							– 0.23	0.056
*R*^2^ change	0.203		0.108		0.100		0.042	

Variables were entered in four different Steps (namely Model 1–4), with each independent variable being assessed for what it might add to the prediction of total hip BMD variance, after the previous variables were controlled for. The relative contribution of each set of variables is also reported (*R*^2^ change). Model 1 included age and BMI as forced entries; Model 2 included HIV related data in addition; Model 3 focused on gonadal function assessment and Model 4, being the final Model, significant at *p* = 0.008, included also VFs as predictor of total hip BMD. Independent variables with the highest significant beta are shown in bold

Abbreviations: *BMI* body mass index, *cART* combined antiretroviral therapy, *TT* total testosterone, *SHBG* sex hormone-binding protein, *cFT* calculated free testosterone, *LH* luteinizing hormone, *FSH* follicle stimulating hormone, *VFs* vertebral fractures

Table 4 shows the results of a backward stepwise logistic regression carried out to assess predictive factors for VFs. Full model included as independent variables age, BMI, years of infection, cART duration, spine BMD, CD4 + nadir count, TT, SHBG, cFT, LH and FSH. At each step, non-significant variables were gradually eliminated to find the best reduced model. Final model (*p* = 0.018) included age, cART duration, TT, SHBG, cFT and FSH, and correctly classified 81.3% of cases. Older age and higher SHBG values were mild predictors of VFs (OR 1.12, *p* = 0.016; OR 1.08, *p* = 0.025, respectively), whereas higher TT levels were protective towards VFs (OR 0.41 *p* = 0.047).

**Table 4:** Results of backward stepwise logistic regression using prevalent VFs as dependent variable

		Prevalent VFs		
	B coefficient	OR (95% CI)	Sig	Excluded variables
Age (years)	0.11	1.12 (1.02–1.23)	0.016	BMI HIV infection duration CD4 nadir Spine BMD LH
cART exposure (years)	0.09	1.10 (0.99–1.20)	0.057	
TT (ng/ml)	– 0.89	0.41 (0.17–0.98)	0.047	
SHBG (nmol/l)	0.08	1.08 (1.01–1.16)	0.025	
cFT (pg/ml)	0.05	1.06 (0.99–1.12)	0.077	
FSH (mUI/ml)	– 0.07	0.93 (0.86–1.01)	0.079	

Abbreviations: *BMI* body mass index, *cART* combined antiretroviral therapy, *TT* total testosterone, *SHBG* sex hormone-binding protein, *cFT* calculated free testosterone, *LH* luteinizing hormone, *FSH* follicle stimulating hormone, *VFs* vertebral fractures

**Fig. 2:**
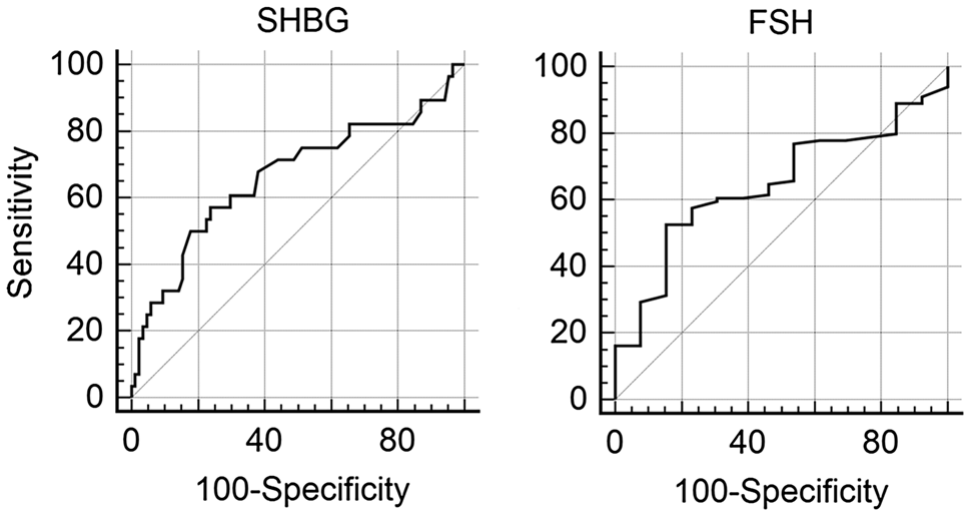
ROC curves with SHBG and VFs, and with FSH and BMD (considered as normal vs osteopenia/osteoporosis) to identify HIV-infected men with skeletal fragility

Finally, ROC curves were calculated to determine whether FSH, TT and SHBG values were predictive of osteopenia/osteoporosis (FSH), and of VFs (TT and SHBG), as shown in Fig. 2. The ROC curves of TT for VFs were not statistically significant, and therefore, not reported. On the contrary, FSH showed a low sensitivity (52.5%) but a good specificity (84.6%) in identifying osteopenic/osteoporotic patients, when threshold value was set at 6.6 IU/l (AUC = 0.645, *p* = 0.039), whereas SHBG showed a moderate sensitivity (57.1%) but a good specificity (76.2%) in identifying patients with VFs, when threshold value was set at 73 mmol/l (AUC = 0.664, *p* = 0.014).

## Discussion

We found a high burden of bone disease in HIV-infected men under chronic cART, being over two thirds of them osteopenic/osteoporotic, and presenting with at least one VF in 25% of cases. Concurrently, we found a high prevalence of gonadal axis alterations, affecting 26.2% of patients. Importantly, we found that the impact of gonadal function on bone health is more evident on VFs than on BMD. In fact, even though some correlations between hormonal profile and BMD were found, we could not build a significant multivariate model able to predict spine BMD. Also, total hip BMD multivariate model showed only partial correlation with gonadal axis, especially with FSH, which had a negative impact. On the contrary, we found a higher prevalence of hypogonadism in patients with VFs, regardless of gonadotropin levels. Furthermore, we found that SHBG was the main factor associated with and predictive for VFs. On the other side, higher TT levels showed some protective role against VFs occurrence.

With respect to BMD data, our findings for osteoporosis (34.5%) and osteopenia (53%) prevalence mainly agree with the literature, even if the prevalence of osteoporosis and osteopenia in HIV-infected men widely varies among studies ranging from 6 to 34% [1] and up to 67% [19, 20], respectively. Of note, a component of iatrogenic hypophosphatemic osteomalacia may be present in these patients, as already reported [21]. Our data on VFs are substantial agreement with literature [22–25], and a recent metanalysis [8] showed a cumulative prevalence of morphometric VFs of 20.2% (CI 15.7–25.6). As for hypogonadism, our findings confirm what already shown in the literature [26–31], especially when considering overt hypogonadism (12.5%).

The impact of hypogonadism on BMD in HIV-infected patients has been investigated by other studies, with partially conflicting results. Whereas some authors showed little or no correlation between BMD and testosterone levels [20, 32–34], in substantial agreement with our findings, others found an association between osteoporosis and hypogonadism [22, 35, 36]. These discrepancies probably come from different study settings, and also from different testosterone assays and cut-off used to define hypogonadism. Furthermore, as suggested by Santi et al. [20], and also supported by Shiyakova. [34], the impact of gonadal function on BMD is mainly mediated by oestradiol, which showed protective role for bone health in men with and without HIV infection. Indeed, we found that also higher levels of FSH and SHBG had a negative impact on BMD. FSH correlated with hip BMD and T-score, and FSH levels > 6.6 mUI/ml represented a good cut-off in predicting osteopenia/osteoporosis. One possible explanation is that an elevated FSH might be a more sensitive marker of testicular dysfunction than a single point testosterone [37]. Furthermore, and more importantly, although it is still a debatable issue, in the last few years accumulating evidence showed that FSH can directly impact on bone, by increasing osteoclastogenesis [38]. Hsu et al. [39] found that higher FSH levels were associated with increased bone loss at the hip over 5 years in elderly men. Moreover, Jing et al. [40] showed that FSH increase was associated with higher risk of osteoporosis/osteopenia in type 2 diabetic men. We previously showed that elevated SHBG levels are very common in HIV-infected men [18, 41] and might represent the *primum movens* of the compensated form of hypogonadism in these patients. In fact, an increase in SHBG would lower the amount of free testosterone and oestradiol, with subsequent compensatory increase in LH production from the pituitary gland, which is able to maintain the testicular production of testosterone. Here, we found that SHBG was negatively associated with spine, hip and femoral BMD and the presence of osteopenia/osteoporosis and that it was positively associated with VFs.

Data on the impact of hypogonadism on fractures in HIV-infected men are more sparse [42], and very rarely hypogonadism was assessed. Among the few studies that evaluated the possible correlation between hypogonadism and fractures in HIV-infected patients, Borderi et al*.* [25] found that hypogonadism was not associated with morphometric VFs. However, they included both men and women, and did not specify how the diagnosis of hypogonadism was done. Short et al*.* [33] found that only the diagnosis of osteoporosis was associated with fractures in HIV-infected men, whereas other parameters, including hypogonadism, were not. However, they considered only self-reported fractures, and did not perform morphometric analysis, which may lead to underdiagnosis, being 65–75% of fragility fractures clinically “silent” [43]. Moreover, they used radioimmunoassay to determine free testosterone, which is unreliable [5]. Indeed, we found that normal testosterone levels were protective towards VFs, whereas higher SHBG values and older age were risk factors. Furthermore, we identified a possible threshold of SHBG (> 73 nmol/L), over which VFs might be more often observed. The role of SHBG in predicting fractures in the general population has also been investigated in few studies. However, a recent meta-analysis by Hidayat et al*.* [44] showed that higher SHBG levels are associated with an increased risk of hip, vertebral and non-vertebral fractures in older adults, both men and women. In addition, high SHBG predicted incident VFs in elderly men and added information for VFs risk prediction beyond the widely used Fracture Risk Assessment Tool (FRAX) [45].

The major limitations of our study include the retrospective nature of the study and the absence of a control group of non-HIV patients. Furthermore, oestradiol, useful to better investigate gonadal function and its relationship with bone health, was not measured in many patients, and we did not collect information to clearly quantify dietary calcium and protein intake. Notwithstanding that, the strengths of our study include the detailed evaluation of hypogonadism, based on clinical evaluation and a complete hormonal assay, as suggested by most guidelines. Furthermore, we focused on the relationship between VFs and hypogonadism as a primary endpoint, a focus very rarely analysed in HIV patients, reporting novel and interesting findings.

In conclusion, we found a high burden of bone and gonadal disease in HIV-infected men under cART, and we showed that, in the multifactorial set of elements involved in the pathogenesis of bone fragility, besides low testosterone levels, FSH might have a negative impact on hip BMD, and, more importantly, higher SHBG values might be predictive for VFs occurrence in this population.

## Data Availability

Data are available upon motivate requests.
